# Trends and drivers of change in the prevalence of anaemia among 1 million women and children in India, 2006 to 2016

**DOI:** 10.1136/bmjgh-2018-001010

**Published:** 2018-10-19

**Authors:** Phuong Hong Nguyen, Samuel Scott, Rasmi Avula, Lan Mai Tran, Purnima Menon

**Affiliations:** 1 Poverty, Health, and Nutrition Division, International Food Policy Research Institute (IFPRI), Washington, District of Columbia, USA; 2 FHI360, Hanoi, Vietnam

**Keywords:** anaemia, child health, maternal health, nutrition, public health

## Abstract

**Introduction:**

India carries the largest burden of anaemia globally. Progress to reduce anaemia has been slow despite substantial economic growth and 50 years of programmatic efforts. Identification of the factors that contribute to anaemia reductions is needed to accelerate progress. We examined changes in haemoglobin (Hb) and anaemia among women and children in India from 2006 to 2016 and identified drivers of changes in these outcomes over time.

**Methods:**

We used two rounds of National Family Health Survey data collected in 2005–2006 and 2015–2016 (n=245 346 children 6–59 months; 37 165 pregnant women (PW) 15–49 years; 760 460 non-pregnant women (NPW) 15–49 years). We first examined trends in Hb and anaemia, and changes in 30 selected variables (including immediate and underlying determinants, and nutrition and health interventions (NHIs)). We identified drivers of Hb and anaemia using multivariate regression and estimated their contribution to changes in these outcomes over time using regression-based decomposition.

**Results:**

Hb and anaemia improved significantly between 2006 and 2016 in children (4.5  g/L and 11 percentage points (pp), respectively) and PW (3.2  g/L and 7.6 pp), but not in NPW. Despite these changes, anaemia is still very high (>50%) and progress varied considerably by state (−33 pp to +16 pp). Most immediate and underlying determinants, and NHIs improved significantly over time. Changes among a set of drivers common to children and PW accounted for the changes in Hb; these included maternal schooling (children, 10%; PW, 24%), coverage of NHIs (children, 18%; PW, 7%), socioeconomic status (children, 7%; PW, 17%), sanitation (children, 3%; PW, 9%), and meat and fish consumption (children, 3%; PW, 1%). The decomposition models moderately explained Hb changes over time (children, 49%; PW, 66%).

**Conclusions:**

Multiple common drivers have contributed to the anaemia changes among children and pregnant women in India. Further improvements in these drivers can have population-level effects by simultaneously influencing both maternal and child anaemia.

**Key questions**

**What is already known?**

- Anaemia affects approximately 2.36 billion individuals globally; progress in reducing anaemia has been slow and evidence on factors explaining changes in anaemia prevalence over time is scarce.

**What are the new findings?**

- From 2006 to 2016 in India, anaemia declined but remained highly prevalent in children and pregnant women; in non-pregnant women, little progress was made.
- The key drivers of anaemia reduction for children were improved health and nutrition interventions, and for pregnant women were improved education and wealth.
- Barriers to progress included low coverage of interventions during pregnancy and early childhood, vegetarian diets, illiteracy and poor sanitation.

**What do the new findings imply?**

- Tackling anaemia in India requires investments in women’s education and socioeconomic status along with continued focus on improving health and nutrition.

## Introduction

Anaemia affects an estimated 2.36 billion individuals globally,^1^ especially women and children.^2^ South Asia accounts for the largest number of anaemia cases, with poor progress being underscored by persistent gaps in coverage of essential nutrition interventions from conception to the child’s second birthday^3^ among other factors. To address the high anaemia burden, the World Health Assembly set a target of achieving a 50% reduction of anaemia in women of reproductive age by 2025 relative to 2010 levels,^4^ but no South Asian country is on track to meet this target.^5^ India, despite a long history of policy commitment and programmes to combat anaemia in children and women,^6^ has not seen much improvement. According to data from the second and third rounds of India’s National Family Health Survey (NFHS) in 1999^7^ and 2006,^8^ anaemia prevalence increased from 74% to 79% in children aged 6 to 36 months and from 52% to 56% in women aged 15 to 49 years. The lack of anaemia reduction is surprising given India’s rapid economic growth^9^ during the same period, as anaemia rates are expected to decline approximately a quarter as fast as income increases.^10^

A recent multicountry survey study estimated that severe anaemia during pregnancy was associated with a twofold risk of maternal mortality.^11^ However, the consequences of anaemia extend beyond death due to severe anaemia. The intangible cost of anaemia—encompassing a range of sequalae such as motor and mental impairment in children and lower work productivity in adults—has been estimated at 1.3% of gross domestic product for children^12^ and 4% of gross domestic product for children and adults combined.^13^ In India, iron deficiency anaemia is one of the top five leading causes of years of life lost due to disability,^5^ thus India is paying heavily for failing to address this severe public health problem. Additional direct causes of anaemia include low absorptive capacity of other micronutrients—especially folic acid and vitamin B_12_—due to intestinal inflammation caused by disease (including obesity) or illness, blood loss and inherited genetic disorders.^14 15^

To accelerate reductions in anaemia, evidence is needed around factors at individual, household and community levels that explain changes in anaemia prevalence over time. Several studies in India have examined cross-sectional associations between anaemia and its ‘determinants’,^16–19^ but very few studies have used multiple rounds of data to model the impact of changes in selected factors on changes in anaemia prevalence over time.^20 21^ Given scarce evidence, policy-makers are challenged to understand which investments will have the greatest impact on future anaemia reduction in India. The traditional approach of iron supplementation and iron fortification of foods^22 23^ may fall short given that only 25%^15^ to 50%^14^ of anaemia is thought to be due to iron deficiency.

The current paper seeks to (1) examine changes in haemoglobin and anaemia among women and children in India from 2006 to 2016 at national and state levels, and (2) identify drivers of haemoglobin and anaemia and estimate their contribution to changes in these outcomes over time.

## Methods

### Data sources

We used nationally representative data from the 2005–2006 and 2015–2016 National Family Health Surveys—NFHS-3^8^ and NFHS-4^24^—conducted by the International Institute for Population Sciences under the stewardship of the Ministry of Health and Family Welfare, Government of India. NFHS-3 included data from 109 041 households and was representative at the state level. NHFS-4 was the first national nutrition survey to be representative at both state and district levels, with data from 601 509 households.

Detailed survey sampling procedures and questionnaires are available in the final reports of NFHS-3^8^ and NFHS-4.^24^ Briefly, both of these surveys used a two-stage sample design stratified by the urban and rural samples within each state. The first stage involved selection of primary sampling units which were villages in rural areas and Census Enumeration Blocks in urban areas. Within each stratum, villages or Census Enumeration Blocks were selected from the sampling frame with probability of selection being proportional to population size. The second stage involved the random selection of 22 households from each primary sampling unit, where a complete household mapping and listing operation was conducted prior to the main survey. Among the selected households, all women aged 15–49 years and children aged 6–59 months were eligible for haemoglobin (Hb) measurement. All individuals with observations for Hb were included in the current analyses: 107 397 and 653 063 non-pregnant women, 5317 and 31 848 pregnant women (gestational age range: 1–9 months), and 35 851 and 209 495 children from NFHS-3 and NFHS-4, respectively, over 1 million women and children, in total.

### Variable identification and construction

The outcomes of interest were Hb level and anaemia status. Hb concentrations were measured from capillary blood samples (by finger prick, or a heel prick for children age 6–11 months) by health investigators, using a portable HemoCue Hb 201+ analyser. Hb level was adjusted for cigarette smoking (in women) and for altitude in enumeration areas that are above 1000 m.^25^ We defined anaemia as a Hb value <120 g/L for non-pregnant women or <110 g/L for pregnant women and children as per WHO norms.^26^

We used the Unicef^27^ and the Lancet Nutrition Series^28^ conceptual frameworks to select the potential drivers of changes in Hb and anaemia including three sets of drivers—immediate determinants, health and nutrition interventions, and underlying determinants.

Immediate determinants included indicators related to maternal undernutrition, diets and disease burden. Women’s low Body Mass Index (BMI<18.5 kg/m^2^) and women’s anaemia (for the model predicting child Hb only) were used to represent maternal undernutrition. Given that child dietary data were only available for children <24 months and that child and maternal diets are highly correlated,^29^ maternal diet was used as a proxy for child diet of all children 6–59 months. Maternal food consumption in the NFHS surveys was measured by asking women how often they consume various foods (daily, weekly, occasionally or never). For the current analyses, two binary indicators were constructed to represent diets: consuming dark green leafy vegetables daily and consuming meat and fish at least once per week. Child diseases included diarrhoea and acute respiratory infection (ARI), which were assessed based on maternal recall of symptoms in the 2 weeks prior to the survey. Diarrhoea was defined as three or more loose stools in a 24-hour period,^30^ and ARI was defined as the presence of cough or cold with fever.^31^

Nutrition and health interventions across the continuum of care from pregnancy to early childhood were selected as a second set of drivers. Interventions during pregnancy included adequate antenatal care (ANC) (at least four ANC visits), iron and folic acid (IFA) supplementation (at least 100 IFA tablets reported to be consumed during the last pregnancy), deworming and weight monitoring. Interventions during early childhood included full immunisation, vitamin A supplementation, paediatric IFA supplementation and deworming. We also examined indicators related to receiving services under India’s Integrated Child Development Services (ICDS) programme (food supplementation, health check-up, and health and nutrition education) during pregnancy, lactation and early childhood. Household-level bednet use was also included.

Underlying determinants were examined at household and maternal levels. At the household level, variables included the number of children under 5 years of age, household socioeconomic status (SES) index, sanitation, religion, place of residence (rural/urban) and scheduled caste/tribe (designated groups of historically disadvantaged persons in India). The SES index was constructed using a principal component extracted from multiple variables including household ownership of 15 assets (car, motorbike, bicycle, television, computer, refrigerator, mobile phone, watch, fan, bed, mattress, table, chair, pressure cooker, sewing machine), livestock (cow, goat, chicken), house and land, as well as key housing characteristics (housing materials for floor, roof, wall and source of cooking).^32^ The first component derived from the component scores explained 62% of the variance and was scaled with the range 0–10 to obtain a measure of household wealth relative to other households, with a higher score indicating higher wealth. The sanitation variable included improved sanitation facility and safe stool disposal. At the maternal level, education and age at first birth were included. All models also adjusted for maternal age, child age and child sex (for childhood anaemia).

### Data analysis

All analyses were conducted separately for children aged 6–59 months, pregnant women (aged 15–49 years) and non-pregnant women of reproductive age. To examine change in Hb by age and across time points, Hb levels were plotted against age (or gestational age for pregnant women). To examine state-level variability in trends of anaemia prevalence, we created maps showing the prevalence in each year as well as the percentage point change from 2006 to 2016. Seven union territories (A&N islands, Chandigarh, D&N Haveli, Daman and Diu, Lakshadweep, Nagaland and Pondicherry) were not included in the 2006 sample frame; therefore, they are not included in the 2006 maps and in further decomposition analyses. Changes in determinants from 2006 to 2016 were tested using a Wald test from linear regression models, adjusted for sampling weights due to complex survey design. Associations between potential determinants and outcomes were examined using multivariate linear (for Hb) or logistic (for anaemia) regression for each survey round separately, and then by pooling data from both rounds. Given minor differences between time points, only pooled results are reported. Place of residence (rural/urban) was dropped from regression analysis because it was highly correlated with other variables (SES, education, etc).

A regression-decomposition analysis was performed to assess how much the change in each determinant contributed to the change in Hb or anaemia prevalence from 2006 to 2016. This analysis combines the analysis of differences in means of the explanatory variables (X) between 2006 and 2016 and regression estimates of the coefficients associated with these variables (Β_X_) from a pooled regression model. For example, if a determinant has a large regression coefficient (‘marginal effect’) and a large change in its mean over time, then this determinant will play a large role in explaining change in Hb level or anaemia prevalence over time. In contrast, if a determinant has either a small regression coefficient or small change over time, the decomposition analysis will not identify the determinant as an important driver of anaemia reduction. Regression-decomposition has been used widely to study mean outcome differences between groups,^33^ including differences in child malnutrition between geographical areas^34–36^ and between populations measured at different points of time.^37^ Because changes in Hb and anaemia were minimal for non-pregnant women, the decomposition analyses were conducted only for children and pregnant women.

All analyses were performed using Stata V.15.1. All regression models were adjusted for the cluster sampling design and sampling weights used in the survey.

## Results

**Figure 1:**
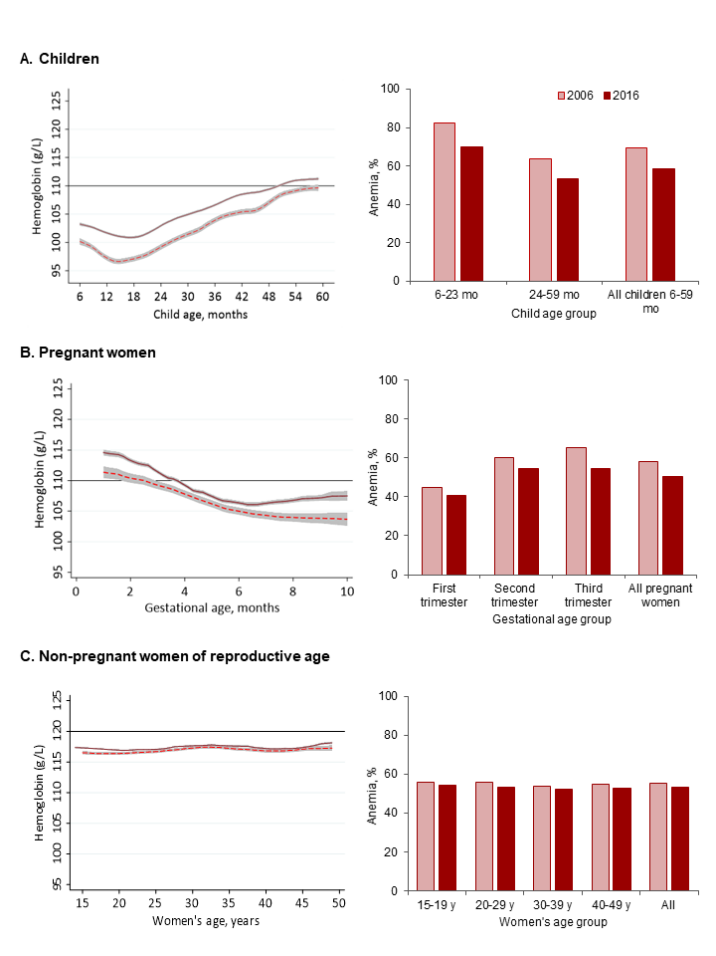
Haemoglobin and anaemia trends in India in 2006 and 2016. For non-pregnant women and children, Hb and anaemia trends are shown by age. For pregnant women, Hb and anaemia trends are shown by gestational age. Anaemia was defined as <110 g/L in children and pregnant women, and <120 g/L in non-pregnant women of reproductive age. Smoothed trends with shaded 95% CIs are shown for 2006 (dotted line) and 2016 (solid line). The horizontal reference line indicates the age-appropriate WHO anaemia cut-off. Data are from the third (2006) and fourth (2016) rounds of India’s National Family Health Survey.

On average, mean Hb improved moderately from 2006 to 2016 in children (change over time=4.5 g/L, 95% CI 4.17 to 4.84; p<0.001) and pregnant women (3.2 g/L, 95% CI 2.53 to 3.79; p<0.001). The improvement in non-pregnant women (1.2 g/L, 95% CI 0.92 to 1.47; p<0.001) was negligible but statistically significant, likely due to the large sample size (figure 1). In both rounds, mean Hb in children declined between 6 and ~14 months of age, followed by a rapid increase from 14 to 59 months of age (figure 1). The mean Hb in children 6 months of age was greater in 2016 than 2006 (103.2 vs 100 g/L) and remained higher at 59 months (109.5 vs 111.2 g/L). In 2016, children on average crossed the anaemia threshold at ~51 months of age, whereas in 2006 they still had not attained non-anaemic status by 59 months. Similar to children, mean Hb among pregnant women was higher at the beginning (114.5 vs 111.3 g/L) and end of gestation (107.8 vs 103.8 g/L) in 2016 compared with 2006 (figure 1). Further, whereas mean Hb continued to decline after 6 months of gestation in 2006, a notable rebound occurred in the third trimester in 2016.

In 2006, 70% of children, 58% of pregnant women and 55% of non-pregnant women were anaemic (figure 1). From 2006 to 2016, anaemia reductions were moderate in children (11 percentage points (pp)) and pregnant women (7.6 pp), but small in non-pregnant women (2.1 pp). Despite these improvements, anaemia was still a severe public health problem in 2016 for all groups according to WHO criteria (>40%): children aged 6–23 months (70%), children aged 24–59 months (53%), pregnant women (50%) and non-pregnant women (53%). Anaemia co-occurred in a third of mothers and children in the same households (data not shown), and more than 95% of anaemia cases were classified as mild or moderate.

**Figure 2:**
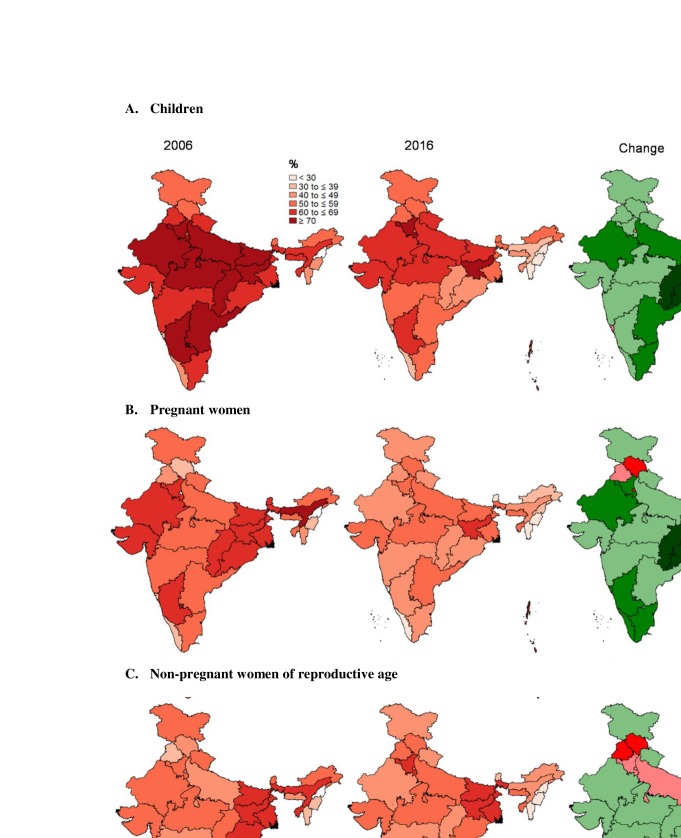
Anaemia prevalence in 2006 and 2016 and changes in anaemia by state. Seven union territories (A&N islands, Chandigarh, D&N Haveli, Daman and Diu, Lakshadweep, Nagaland and Pondicherry) were not included in the 2006 sample frame; therefore, they are not included in the 2006 maps. Anaemia was defined as <110 g/L in children and pregnant women, and <120 g/L in non-pregnant women of reproductive age. Data are from the third (2006) and fourth (2016) rounds of India’s National Family Health Survey. pp, percentage point.

The rate of anaemia reduction varied by state: −33 pp to +10 pp for children, −38 pp to +16 pp for pregnant women, and −24 pp to +16 pp for non-pregnant women (figure 2 and online supplementary table 1). For children, the most improvement was seen in Assam, where anaemia was nearly halved, from 69% to 36%, followed by Chhattisgarh, with a 30 pp decline (figure 2 and online supplementary table 1). In contrast, anaemia prevalence among children in Goa increased by 9 pp. Among pregnant women, Sikkim showed the most improvement with a 38 pp reduction, followed by Assam (27 pp decline) and Mizoram (21 pp decline). Though the national average for anaemia among non-pregnant women did not change much in the last decade, some states did show noteworthy gains (figure 2). For example, in Sikkim and Assam, anaemia in non-pregnant women fell by more than 20 pp. In contrast, the problem worsened in states such as Himachal Pradesh and Punjab, where anaemia in non-pregnant women increased by more than 10 pp. Overall, anaemia prevalence declined in most states, but increased in two states for children, three states for pregnant women and eight states for non-pregnant women.

10.1136/bmjgh-2018-001010.supp1 Supplementary data

**Table 1:** Prevalence of selected factors hypothesised to be associated with anaemia among children, pregnant women and non-pregnant women in India in 2006 and 2016

Anaemia drivers	Children (n=245 346)		Pregnant women (n=37 165)		Non-pregnant women (n=760 460)	
	2006	2016	2006	2016	2006	2016
Immediate determinants						
Women’s low BMI, %	41.8	26.3***	21.5	14.0***	35.7	22.9***
Maternal anaemia, %	60.1	57.1***	–	–	–	–
Child diarrhoea, %	9.2	9.1	–	–	–	–
Child ARI, %	5.9	2.8***	–	–	–	–
Women’s daily meat and fish consumption, %	5.7	5.0	5.7	5.1	7.0	6.1*
Women’s weekly meat and fish consumption, %	31.7	41.0***	31.6	39.8***	35.7	42.6***
Women’s daily dark green vegetable consumption, %	61.6	46.4***	64.1	47.5***	64.1	47.2***
Nutrition and health interventions†						
At least 4 ANC visits, %	33.6	48.2***	27.8	41.8***	37.9	51.7***
IFA consumption 100 tablets, %	14.3	29.0***	12.9	25.8***	16.0	30.9***
Deworming during pregnancy, %	3.6	17.7***	3.0	14.9***	3.8	18.4***
Weighed during pregnancy, %	45.9	73.3***	39.4	68.1***	50.0	76.0***
Paediatric full immunisation, %	41.6	55.7***	–	–	–	–
Paediatric vitamin A supplementation, %	15.1	57.9***	–	–	–	–
Paediatric IFA, %	4.5	25.9***	–	–	–	–
Paediatric deworming, %	12.2	31.8***	–	–	–	–
ICDS‡—food supplementation, %	31.1	63.6***	17.3	53.7***	–	–
ICDS‡—health check-up, %	20.3	55.4***	11.0	44.4***	–	–
ICDS‡—health and nutrition education, %	21.6	54.5***	8.5	38.6***	–	–
Using bednet, %	41.3	39.1*	41.2	37.6**	38.6	34.3***
Underlying determinants						
Household size, n	6.7	6.4***	6.0	5.7***	5.9	5.6***
No. of children <5 years, n	1.6	1.6	0.8	0.7	0.4	0.3
Household SES index, n	3.9	5.7***	3.5	5.3***	4.0	5.6***
Improved sanitation facilities, %	22.3	40.1***	22.1	42.1***	31.7	50.3***
Safe stool disposal, %	18.6	34.2***	14.5	30.8***	21.0	36.1***
Scheduled caste/tribe, %	71.4	76.5***	71.4	76.5***	66.8	73.2***
Hindu religion, %	78.7	78.4	78.8	77.3	81.2	80.5
Muslim religion, %	16.6	16.7	17.2	17.6	13.0	13.8
Rural, %	76.1	72.5**	76.6	72.6**	68.4	66.2
Maternal schooling, years	4.0	6.3***	4.4	7.2***	5.0	6.8***
Mothers with no schooling, %	50.0	30.7***	46.6	25.0***	40.6	28.0***
Mothers with ≥10 years of schooling, %	16.0	30.3***	17.7	36.2***	21.9	35.2***
Married before 18 years, %	60.9	38.9***	56.6	32.6***	60.8	45.3***
Maternal age, years	26.7	27.2***	23.5	24.3***	29.4	30.2***
Other factors						
Male child, %	52.9	52.2*	–	–	–	–
Child aged 6–23 months, %	32.6	32.9				
Child aged 24–59 months, %	67.4	67.1	–	–	–	–

Significant changes from 2006 to 2016 were tested using the Wald test from linear regression models, adjusted for sampling weights: ***p<0.001, **p<0.01, *p<0.05.

†Variables related to the most recent pregnancy.

‡ICDS—food supplementation, health check-up, and health and nutrition counselling in children model includes any of the services during pregnancy, lactation or during early childhood.

ANC, antenatal care; ARI, acute respiratory infection; BMI, Body Mass Index; ICDS, Integrated Child Development Services; IFA, iron and folic acid; SES, social economic status.

The improvements in Hb and anaemia among children and pregnant women were paralleled by modest and mixed improvements in the immediate determinants, nutrition and health interventions, and underlying factors (table 1). Low BMI among mothers of children aged 6–59 months declined by 16 pp. The disease burden among children improved for ARI but remain unchanged for diarrhoea. Women’s daily meat and fish consumption remained low at 5%, but weekly meat and fish consumption increased by 8.2 pp, such that 40% of women in 2016 reported consuming meat and fish at least once per week. However, daily dark green vegetable consumption among women declined by 16.6 pp.

As shown in table 1, all nutrition and health interventions across the continuum of care improved over time, except for the proportion of households reporting bednet use, which fell slightly. For example, the percentage of mothers receiving four ANC visits increased by 14 pp, consumption of IFA supplements during pregnancy doubled from 12.9% to 25.8% and deworming during pregnancy increased by 11.9 pp. Improvement was also observed for several child-level interventions, that is, immunisation, vitamin A and IFA supplementation, deworming and ICDS service use. There were also economic and social changes from 2006 to 2016, especially in relation to household SES, improved sanitation facilities, safe stool disposal, women’s education, age at marriage and urbanisation.

In multivariate regression analyses using data pooled from both survey rounds, significant associations were found between several determinants and child and women outcomes (table 2). Among immediate determinants, women’s low BMI (β=−0.68), women’s anaemia (β=−4.51) and child diarrhoea (β=−1.24) were negatively associated with child Hb while women’s consumption of meat and fish as well as dark green leafy vegetables showed a positive association (β=1.52 and β=0.25, respectively). Among nutrition and health interventions, six interventions positively associated with child Hb were IFA consumption during pregnancy (β=0.31), deworming during pregnancy (β=0.44), weight monitoring during pregnancy (β=1.16), paediatric deworming (β=0.34), receiving food supplementation during pregnancy, lactation or early childhood (β=0.51), and use of bednets (β=1.67). Among underlying determinants, child Hb was positively associated with household SES, improved sanitation facilities, safe stool disposal and maternal schooling, and negatively associated with total number of children in the household aged <5 years, scheduled caste/tribe, and Hindu or Muslim religion. Similar associations were observed when predicting Hb in pregnant women (except for IFA consumption, weight monitoring during pregnancy and use of bednets). Among non-pregnant women, due to the interaction between BMI and the consumption of meat and fish, we stratified analyses by BMI status (online supplementary table 2). The magnitude of association between meat and fish consumption with Hb was much larger (β=0.84) among women with low BMI (<18.5 kg/m^2^) compared with those with normal BMI (18.5–24.9 kg/m^2^) (β=0.25) or who were overweight or obese (≥25 kg/m^2^) (β=0.37). When using anaemia as the outcome instead of Hb, a similar pattern of associations was observed, with coefficients in the opposite direction given the inverse association between Hb and anaemia (online supplementary table 3).

10.1136/bmjgh-2018-001010.supp2 Supplementary data

10.1136/bmjgh-2018-001010.supp3 Supplementary data

**Table 2:** Associations between haemoglobin and selected factors among children, pregnant women and non-pregnant women in India using pooled data from 2006 and 2016

Anaemia drivers	Children (n=2 45 346)		Pregnant women (n=37 165)		Non-pregnant women (n=7 60 460)	
	β	95% CI	β	95% CI	β	95% CI
Immediate determinants						
Women’s low BMI	−0.68***	−0.88 to 0.47	−0.50	−1.15 to 0.14	−2.88***	−3.05 to 2.71
Maternal anaemia	−4.51***	−4.70 to 4.32	–	–	–	–
Child diarrhoea	−1.24***	−1.57 to 0.92	–	–	–	–
Child ARI	−0.28	−0.78 to 0.22	–	–	–	–
Women’s weekly meat and fish consumption	1.52***	1.29 to 1.74	0.45+	−0.03 to 0.94	−0.32***	−0.48 to 0.15
Women’s daily dark green vegetable consumption	0.25*	0.04 to 0.46	0.36	−0.09 to 0.81	0.18*	0.02 to 0.33
Nutrition and health interventions						
At least 4 ANC visits	−0.11	−0.35 to 0.12	−0.22	−0.96 to 0.51	–	–
IFA consumption 100 tablets	0.31*	0.07 to 0.56	0.34	−0.45 to 1.13	–	–
Deworming during pregnancy	0.44**	0.14 to 0.73	1.61**	0.61 to 2.61	–	–
Weighed during pregnancy	1.16***	0.91 to 1.41	−0.49	−1.17 to 0.19	–	–
Paediatric full immunisation	−0.01	−0.21 to 0.19	–	–	–	–
Paediatric vitamin A supplementation	0.17	−0.06 to 0.39	–	–	–	–
Paediatric IFA	−0.06	−0.33 to 0.21	–	–	–	–
Paediatric deworming	0.34**	0.09 to 0.58	–	–	–	–
ICDS—food supplementation	0.51***	0.22 to 0.80	0.07	−0.75 to 0.89		
ICDS—health check-up	0.22	−0.10 to 0.54	0.05	−0.97 to 1.07	–	–
ICDS—health and nutrition education	−0.18	−0.50 to 0.15	−0.95	−1.93 to 0.02	–	–
Using bednet	1.67***	1.45 to 1.88	−0.33	−0.79 to 0.13	−0.69***	−0.86 to 0.53
Underlying determinants						
No. of children <5 years	−0.65***	−0.81 to 0.50	−1.37***	−1.80 to 0.95	−1.10***	−1.20 to 1.00
Household SES index (0–10), score	0.17***	0.12 to 0.21	0.30***	0.20 to 0.40	0.17***	0.14 to 0.21
Improved sanitation facilities	0.50***	0.24 to 0.76	0.63*	0.06 to 1.20	0.27**	0.09 to 0.44
Stool safe disposal	0.30*	0.05 to 0.55	0.94*	0.11 to 1.78	0.42**	0.10 to 0.74
Scheduled caste/tribe	−0.44***	-0.71 to 0.18	−0.98***	−1.55 to 0.40	−0.73***	−0.91 to 0.55
Hindu religion	−0.62**	−1.07 to 0.17	−0.56	−1.53 to 0.41	−0.54**	−0.88 to 0.21
Muslim religion	−1.05***	−1.59 to 0.51	−0.26	−1.38 to 0.86	0.68***	0.29 to 1.08
Maternal schooling, years	0.20***	0.18 to 0.23	0.27***	0.22 to 0.33	0.10***	0.09 to 0.12
Married before 18 years	−0.03	−0.23 to 0.17	−0.12	−0.61 to 0.38	−0.32***	−0.46 to 0.18
Maternal age, years	0.07***	0.05 to 0.09	0.07*	0.01 to 0.12	−0.02***	−0.03 to 0.01
Other factors						
Male	−0.28**	−0.45 to 0.11	–	–	–	–
Child aged 24–59 months	5.81***	5.61 to 6.00	–	–	–	–
Months of pregnancy	–	–	−1.08***	−1.18 to 0.97	–	–

P values were obtained from multivariate linear regression models, adjusted for sampling weights: ***p<0.001, **p<0.01, *p<0.05, +p<0.10.

ANC, antenatal care; ARI, acute respiratory infection; BMI, Body Mass Index; ICDS, Integrated Child Development Services; IFA, iron and folic acid; SES, socioal economic status.

**Figure 3:**
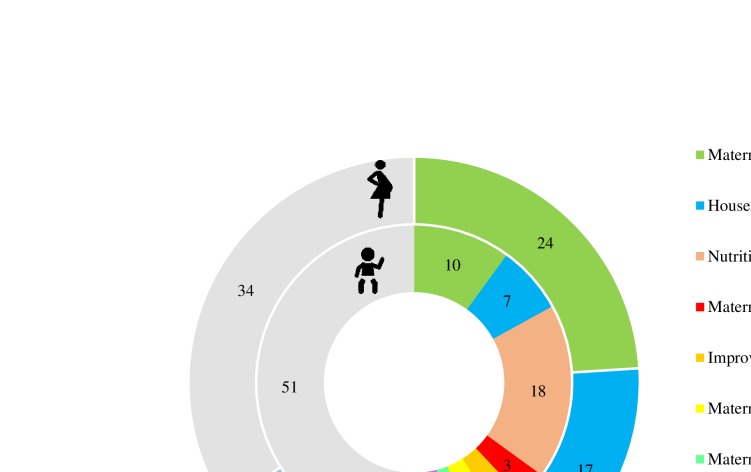
Decomposition analysis for factors contributing to change in haemoglobin among children and pregnant women in India from 2006 to 2016. Results for pregnant women are shown in outer doughnut and results for children in inner doughnut. Numbers indicate the percentage of change in haemoglobin accounted for by the change in the factor. For pregnant women, nutrition and health interventions include deworming and consumption of 100+ IFA tablets during pregnancy. For children, nutrition and health interventions include services for both mothers (deworming and consumption of 100+ IFA tablets during pregnancy, weight monitoring, ICDS during pregnancy and lactation) and children (vitamin A supplementation, paediatric deworming). BMI, Body Mass Index; ICDS, Integrated Child Development Services; IFA, iron and folic acid; SES, socioeconomic status. Data are from the third (2006) and fourth (2016) rounds of India’s National Family Health Survey.

The regression models from table 2 addressed the question of which factors were associated with Hb. The significant variables from the regression analyses were then used in decomposition analyses to estimate the extent to which improvements in these factors contributed to improvements in Hb over time (figure 3). Overall, the decomposition model for children 6–59 months performed moderately, explaining 49% of the Hb change from 2006 to 2016 (figure 3). The explained share was accounted for by the improvements in nutrition and health interventions (18%; among them ANC care, 7%; IFA consumption during pregnancy, 1%; deworming, 1.4%; child vitamin A supplementation, 1.6%; child deworming, 1.5%; child food supplementation through ICDS 3.7%; and child health check-up through ICDS, 1.3%), women’s schooling (10%), SES (7%), and changes in meat and fish consumption, improved sanitation facilities, maternal anaemia and low BMI (each 2%–3%). Similar findings were found in the decomposition analyses for anaemia and separately among children 6–23 and 24–59 months (results not shown).

In pregnant women, the decomposition models explained 66% of the measured difference in Hb between 2006 and 2016 (figure 3). Hb improvements in pregnant women were explained by improvements in maternal schooling (24%), SES (17%), nutrition and health interventions (7%), improved sanitation (9%), number of children <5 years (6%), maternal age (2%), and meat and fish consumption (1%).

## Discussion

Between 2006 and 2016, India made considerable progress in reducing anaemia prevalence among children aged 6–59 months and pregnant women, but not among non-pregnant women. In this paper, we quantified the marginal effects of Hb and anaemia drivers and used the mean changes in these drivers to account for changes in Hb and anaemia over time. We found commonalities in factors contributing to improvement in Hb concentration and anaemia prevalence in children and pregnant women. While improvement in nutrition and health interventions was the strongest driver of anaemia reduction in children, improvements in maternal schooling and SES were the key drivers of anaemia reduction in pregnant women. Higher maternal BMI, greater animal-source food consumption among mothers, improvements in sanitation and fewer young children per household were also important drivers of improvements in Hb and anaemia from 2006 to 2016.

Our findings on determinants of anaemia are well aligned with those from other studies that have examined the determinants of anaemia at global^38^ and regional levels,^5 39^ as well as in India.^16–21 40^ Regarding India, nearly all previous work has found associations between anaemia and maternal education,^16 20 21^ wealth status and caste.^17 18 20 40^ Some studies also link anaemia in India to monotonous diets^16 21^ and poor sanitation,^21^ even after controlling for household SES and women’s education. However, our paper goes well beyond the cross-sectional analyses of associations conducted in previous studies. We used the most recent data from NFHS-4 (2015–2016), merged with the previous NFHS-3 survey (2005–2006), to comprehensively examine the changes in Hb and anaemia after a 10-year gap at both national and state levels, as well as for different age groups and target populations (children, pregnant women and non-pregnant women). More importantly, we applied the decomposition technique using a rich set of available explanatory variables at individual, household and community levels in both 2006 and 2016 NFHS datasets, to comprehensively examine factors contributing to changes in Hb and anaemia over time.

Our analyses indicate that improvements in coverage of nutrition and health interventions substantially contribute to improvements in Hb (18% in children and 7% in pregnant women). This is further supported by observable changes in the distribution of Hb in our sample (figure 1). The upward shift in Hb in pregnant women in their third trimester from 2006 to 2016 is indicative of a reestablishment of biological norms, as anaemia resulting from haemodilution during the second trimester normally rebounds by the end of pregnancy. Whereas in 2006, Hb levels were low even for women at the end of pregnancy, in 2016 the expected higher Hb during the end of pregnancy was seen. Our decomposition results indicate that such a shift could be attributable to improvements in women’s education, socioeconomic status, and health and nutrition interventions during pregnancy. Such improvement would be expected to carry over to children in terms of a greater fetal nutrient endowment, with iron being particularly relevant, which is evident from the higher Hb concentration among children 6 months of age in 2016 compared with 2006. Interventions targeted at pregnant women together with interventions for women and children during childhood—such as ICDS for lactating mothers, paediatric IFA and deworming—could together contribute to anaemia reduction in children 6–59 months. Although these nutrition and health interventions had positive trends in the last decade, coverage remains unacceptably low for some interventions in 2016, such as IFA consumption (30%) and deworming during pregnancy (18%) or early childhood (32%), indicating substantial room for improvement. The ICDS findings, where we saw that the benefit in children was primarily for food supplementation rather than health check-ups or nutrition education, should be interpreted with caution. The food supplementation variable only reflects self-reported receipt of food supplements, not consumption level. Further, the quality of health service delivery is unknown, and low-quality health check-ups and nutrition education, even if received, may underscore the null association with anaemia.

Women’s education, SES and household sanitation were the most important underlying determinants of anaemia reduction. Women’s educational attainment has improved substantially from 2006 to 2016 (the percentage of women with no education decreased by 22 pp in pregnant women and by 13 pp in non-pregnant women; table 1). Improvements in education explained 10% of the overall Hb improvement in children and 24% in women. SES gains were particularly important in contributing to anaemia reduction in pregnant women, explaining 17% of the variance in change. Similarly, presence of an improved sanitation facility at the household level increased by 17–20 pp from 2006 to 2016, a change that accounted for an estimated 3%–9% decline in anaemia over this 10-year period. Despite these improvements, there is significant room for improvement in women’s education, SES and sanitation. As of 2016, nearly a third of Indian women are still illiterate, only ~30% had at least 10 years of schooling and only 50% of households used an improved sanitation facility. Further investments in women’s education, women’s livelihoods and household sanitation are needed to optimally reduce anaemia among women and children.

Changes in immediate determinants accounted for a low percentage of the overall anaemia reduction from 2006 to 2016. Weekly meat and fish consumption increased by 7–9 pp from 2006 to 2016, an increase that accounted for an estimated 1%–3% anaemia reduction after adjusting for covariates such as household SES and women’s education. Given that ~60% of the Indian population does not consume meat and fish, and that even among the non-vegetarian population, meat and fish are consumed sparingly, increased meat and fish consumption would only be a solution in the fraction of the population that consumes and can afford these relatively expensive foods. Dark green leafy vegetables, consumed far more frequently than meat and fish in this population, are a good source of nutrients that may benefit anaemia.^21^ However, the daily consumption of dark green vegetables has reduced substantially (16 pp, from 64% to 48%) in the last decade. Moreover, we could not find evidence to suggest that a large-scale intervention to increase dark green vegetable consumption would be effective in terms of reducing anaemia. Since the diet of this population is largely cereal based, cereal fortification and biofortification are important solutions to anaemia, with special consideration to use modern biotechnological methods to both improve micronutrient content and reduce phytate content in cereals.^41–43^ However, iron fortification of wheat provided through public programmes in India has had limited success in reducing anaemia given implementation challenges, including poor distribution channels and poor targeting.^44^ Newer fortification legislation in India,^45^ which engages a wider network of food manufacturers and the private sector, may have more far-reaching impacts. Both low BMI (undernourished) and high BMI (overweight and obesity) are considered as risk factors for iron deficiency,^46^ one known cause of anaemia. In our study, improvement in maternal low BMI contributed to a small proportion of anaemia reduction in their children (2%). Although overweight and obesity has increased in the last 10 years, the data do not support the notion that increasing BMI is associated with increasing anaemia at the population level. This is likely due to the multifactorial nature of anaemia, with iron deficiency only being one factor. Further, while programmes such as the public distribution system deliver foods that provide energy, and therefore may increase BMI, these same foods are not necessarily dense in other nutrients that would reduce anaemia.

Progress in anaemia reduction has not been uniform across states in India. While positive changes were observed in several states, others showed lack of improvement. Notably, anaemia prevalence has increased for all population groups in Delhi, the densely populated urban capital of India. We suggest that this may be due to urban expansion,^21^ the concomitant influx of low-income migrants and a shifting dietary landscape to foods with low nutrient density, though additional work is needed to support this hypothesis. Although all states operate under a similar broad national policy and programmatic environment, there is high variability in the implementation of nutrition policies and programmes across different states.^47^ The newly launched National Nutrition Strategy^48^ also emphasises decentralisation at state and even district levels. An understanding of variation in each state, considering state-level successes, challenges and needs, can facilitate cross-state learning and identification of state-specific areas for strategic investments to further accelerate anaemia reduction.

Despite the improvement in Hb and reduction in anaemia between 2006 and 2016, anaemia remains a critical concern in India, with more than half of children and pregnant women anaemic in 2016. Perhaps most worrisome is the stagnant problem of anaemia in non-pregnant women of reproductive age. Although anaemia in non-pregnant women shared some common underlying determinants as in children and pregnant women, we were unable to examine anaemia drivers in non-pregnant women because there was almost no change in anaemia in this group during the past decade. We note that although the population prevalence of low BMI in non-pregnant women decreased considerably, there was not a substantial decrease in anaemia. This suggests that women gained weight but did not necessarily experience improvements in micronutrient status or other factors underlying anaemia. Poor progress in non-pregnant women is likely due to inadequate policy focus on this segment of the population.

Most national anaemia prevention programmes have focused exclusively on pregnant women and young children. A few have focused on adolescents, but have likely been ineffective given only a 1.7 pp reduction (from 55.8% to 54.1%) in girls aged 15–19 years in the last decade. Younger adolescents aged 10–14 years are not included in NFHS, thus the national prevalence of anaemia in this age group is unknown. However, recent subnational surveys have shown that between 50% and 90% of adolescents are anaemic during this critical period when lifelong habits are being formed.^16 49 50^ Given that most Indian girls reach menarche (thus start to experience significant iron losses) between age 12 and 14 years^51^ and have their first child by age 19 years,^24^ it is important to intervene early. It is also important to track progress in this group, which necessitates including younger adolescents in national surveys. Finally, in older non-pregnant women of reproductive age who are unlikely to have more children, anaemia reduction is still a high priority given demonstrated associations with factors important in everyday life such as cognitive function and labour productivity.^1^

A few methodological notes are worth mentioning for data interpretation. The decomposition approach combines the analysis of the changes in means of the explanatory variables and regression estimates of the coefficients associated with these variables.^33^ Therefore, the decomposition results do not show factors significantly associated with Hb and anaemia in the regression models but which did not change over time. For example, bednet use was positively associated with Hb, but this practice fell slightly over time (2–4 pp); therefore, it did not contribute to anaemia reduction in the studied period. However, bednet use may still be an important factor to be addressed for the prevention of anaemia in the future. Similarly, dark green vegetable consumption was positively associated with Hb. Since consumption declined over time, it did not contribute to anaemia reduction; however, this does not mean that increasing dark green vegetable consumption would not benefit anaemia.

We acknowledge some other limitations that may account for some of unexplained variance in the decomposition models. Although the NFHS surveys are large, nationally representative and contain rich data on health and nutrition outcomes, several important factors known to be associated with anaemia were not available. For example, the lack of information on infectious disease—such as the intestinal parasites, malaria, tuberculosis and HIV/AIDS—prevented us from being able to examine whether there have been changes in these factors, as well as their contribution to anaemia reduction. Data on genetic Hb disorders, which are prevalent in some developing countries,^38^ were also not available. Such genetic disorders have been found to be negatively associated with Hb in Indian children.^19^ With regards to diet, the NFHS surveys only capture information on food group consumption. Detailed dietary information was not available to further examine the effects of individual nutritional factors although a recent study in India showed that changes in the household supply of iron, folic acid and phytate were significantly associated with changes in anaemia prevalence between 2002–2004 and 2012–2013.^21^

## Conclusion

Despite progress in the last decade for anaemia reduction in children and pregnant women, anaemia continues to be a major public health concern in India. Our findings have revealed multiple common drivers of anaemia reduction among children and pregnant women. To accelerate anaemia reduction, a holistic approach targeting the known underlying determinants of anaemia is needed. It is reassuring that the 2018 Anaemia Mukt Bharat guidelines take a more integrated approach than ever before. Interventions in the guidelines include IFA supplementation alongside behaviour change communication to improve pill-taking compliance, deworming, education on appropriate dietary choices and child feeding practices, promotion of delayed cord clamping, mandatory provision of fortified foods in public health programmes, and screening and treatment of non-nutritional causes of anaemia with a focus on malaria. Our results highlight that a focus is also needed on (1) improving women’s education so they can make better dietary choices for themselves and their families, (2) ensuring access to and timely provision of high-quality health services for women of all socioeconomic strata and castes, (3) creating livelihoods for poor households to boost their economic conditions, and (4) reducing open defecation and implementing behaviour change campaigns to promote hygiene and sanitation in rural communities.

By 2024, India is expected to be the most populated country in the world.^52^ Given the large number of affected individuals in this country, investments in evidence-backed actions to reduce anaemia are important for Indians and are critical to achieve global anaemia reduction targets.
