# Supplementary material for: Trends and drivers of change in the prevalence of anaemia among 1 million women and children in India, 2006 to 2016
Source: BMJ Glob Health. 2018 Oct 19;3(5):e001010. doi: 10.1136/bmjgh-2018-001010 (PMC6202996; doi:10.1136/bmjgh-2018-001010)
Supplement: Supplementary data [file bmjgh-2018-001010supp001.pdf]

**Supplementary table 1: Anemia among children aged 6-59 months, pregnant women and non-pregnant women of reproductive age in India in 2006 and 2016, by state**

| State | Children | | Pregnant women | | Non-pregnant women | |
|---|---|---|---|---|---|---|
| | 2006 | 2016 | 2006 | 2016 | 2006 | 2016 |
| A & N ISLANDS | -- | 50.3 | -- | 61.4 | -- | 65.7 |
| ANDHRA PRADESH | 70.9 | 59.6 | 58.2 | 51.1 | 63.2 | 58.9 |
| ARUNACHAL PRADESH | 58.1 | 54.4 | 51.8 | 37.7 | 50.6 | 43.6 |
| ASSAM | 69.0 | 35.9 | 72.0 | 44.9 | 69.1 | 46.0 |
| BIHAR | 77.9 | 63.5 | 60.2 | 58.3 | 68.2 | 60.5 |
| CHANDIGARH | -- | 72.4 | -- | 76.8 | -- | 76.0 |
| CHATTISGARH | 72.1 | 41.7 | 63.1 | 41.5 | 57.2 | 47.3 |
| D & N HAVELI | -- | 84.3 | -- | 68.0 | -- | 80.1 |
| DAMAN & DIU | -- | 74.6 | -- | 39.3 | -- | 59.4 |
| DELHI | 57.2 | 59.8 | 29.9 | 46.3 | 45.0 | 54.6 |
| GOA | 38.8 | 48.4 | 37.0 | 26.8 | 37.9 | 31.4 |
| GUJARAT | 70.0 | 62.4 | 60.8 | 51.2 | 55.2 | 55.0 |
| HARYANA | 72.5 | 71.8 | 69.7 | 55.0 | 55.2 | 63.1 |
| HIMACHAL PRADESH | 54.0 | 53.6 | 38.1 | 50.4 | 43.2 | 53.6 |
| JAMMU & KASHMIR | 58.9 | 54.2 | 55.7 | 47.4 | 51.9 | 49.5 |
| JHARKHAND | 70.5 | 70.1 | 68.5 | 62.6 | 69.4 | 65.3 |
| KARNATAKA | 70.6 | 61.3 | 60.5 | 45.4 | 50.8 | 44.8 |
| KERALA | 44.6 | 35.9 | 33.8 | 22.6 | 32.8 | 34.7 |
| LAKSHADWEEP | -- | 52.6 | -- | 39.0 | -- | 46.3 |
| MADHYA PRADESH | 73.9 | 69.0 | 57.9 | 54.6 | 55.8 | 52.4 |
| MAHARASTRA | 63.5 | 53.9 | 57.8 | 49.3 | 48.1 | 47.9 |
| MANIPUR | 41.6 | 24.0 | 36.2 | 26.0 | 35.6 | 26.5 |
| MEGHALAYA | 64.7 | 48.1 | 58.1 | 53.2 | 45.4 | 56.4 |
| MIZORAM | 44.1 | 19.8 | 48.2 | 27.2 | 37.6 | 24.9 |
| NAGALAND | -- | 26.6 | -- | 32.6 | -- | 27.6 |
| ORISSA | 65.4 | 44.7 | 68.2 | 47.6 | 60.9 | 51.2 |
| PONDICHERRY | -- | 44.9 | -- | 25.9 | -- | 53.3 |
| PUNJAB | 66.2 | 56.6 | 41.6 | 42.0 | 37.9 | 53.9 |
| RAJASTHAN | 70.6 | 60.5 | 61.7 | 46.7 | 52.6 | 46.9 |
| SIKKIM | 57.3 | 55.6 | 62.0 | 23.6 | 59.3 | 35.2 |
| TAMIL NADU | 63.6 | 50.8 | 54.6 | 44.4 | 53.1 | 55.4 |
| TRIPURA | 63.2 | 48.3 | 57.6 | 54.4 | 65.6 | 54.5 |
| UTTAR PRADESH | 74.0 | 63.4 | 51.5 | 51.0 | 49.7 | 52.5 |
| UTTARANCHAL | 61.1 | 60.2 | 50.9 | 46.5 | 54.9 | 45.1 |
| WEST BENGAL | 61.0 | 54.4 | 62.6 | 53.5 | 63.2 | 62.8 |

Data are from the third (2006) and fourth (2016) rounds of India’s National Family Health Survey.
