# Supplementary material for: Trends and drivers of change in the prevalence of anaemia among 1 million women and children in India, 2006 to 2016
Source: BMJ Glob Health. 2018 Oct 19;3(5):e001010. doi: 10.1136/bmjgh-2018-001010 (PMC6202996; doi:10.1136/bmjgh-2018-001010)
Supplement: Supplementary data [file bmjgh-2018-001010supp002.pdf]

Supplementary Table 2. Associations between hemoglobin and selected factors among non-pregnant women in India, stratifying by BMI status, using pooled data from 2006 and 2016

	BMI < 18.5 kg/m^2 (N=177,526)		18.5 ≤ BMI < 25 kg/m^2 (N=444,160)		BMI ≥ 25 kg/m^2 (N=136,159)	
**Anemia drivers**	**β**	**95% CI**	**β**	**95% CI**	**β**	**95% CI**
**Immediate determinants**						
Women's weekly meat and fish consumption	0.84***	0.52,1.15	0.25*	0.05,0.45	0.37*	0.07,0.68
Women's daily dark green vegetable consumption	0.16	-0.14,0.46	0.10	-0.09,0.28	0.37*	0.05,0.69
**Nutrition and health interventions**						
Using bednet	-0.12	-0.42,0.18	-0.69***	-0.88,-0.49	-0.89***	-1.21,-0.57
**Underlying determinants**						
Number of children <5y	-0.70***	-0.90,-0.51	-1.27***	-1.40,-1.14	-0.69***	-0.97,-0.40
Household SES index (0-10), score	0.16***	0.08,0.23	0.16***	0.12,0.20	0.08*	0.02,0.14
Improved sanitation facilities	0.18	-0.21,0.57	0.33**	0.11,0.55	-0.35*	-0.70,-0.01
Stool safe disposal	-0.25	-0.90,0.41	0.19	-0.18,0.56	0.41	-0.11,0.93
Scheduled caste/tribe	-0.67**	-1.07,-0.27	-0.64***	-0.85,-0.42	-0.90***	-1.22,-0.58
Hindu religion	0.75+	-0.06,1.55	-0.59**	-0.98,-0.19	-0.90**	-1.44,-0.36
Muslim religion	1.98***	1.07,2.89	0.53*	0.05,1.01	0.04	-0.60,0.68
Maternal schooling, y	0.14***	0.10,0.18	0.13***	0.10,0.15	-0.04*	-0.08,-0.01
Married before 18 y	-0.19	-0.49,0.11	-0.35***	-0.53,-0.17	-0.59***	-0.90,-0.27
Maternal age, y	-0.07***	-0.09,-0.05	-0.01*	-0.02,-0.00	-0.05***	-0.07,-0.02

BMI, Body mass index; ARI, Acute respiratory infection; ANC, antenatal care; IFA, iron and folic acid; ICDS, Integrated Child Development Services; SES, Social Economic Status. P-values were obtained from multivariate linear regression models, adjusted for sampling weights: ***p<0.001, **p<0.01, *p<0.05, +p<0.10
