# Supplementary material for: Trends and drivers of change in the prevalence of anaemia among 1 million women and children in India, 2006 to 2016
Source: BMJ Glob Health. 2018 Oct 19;3(5):e001010. doi: 10.1136/bmjgh-2018-001010 (PMC6202996; doi:10.1136/bmjgh-2018-001010)
Supplement: Supplementary data [file bmjgh-2018-001010supp003.pdf]

Supplementary Table 3. Associations between anemia and selected factors among children, pregnant women, and non-pregnant women in India using pooled data from 2006 and 2016

	Children (n = 245,346)		Pregnant women (n = 37,165)		Non-pregnant women (n = 760,460)	
**Anemia drivers**	**OR**	**95% CI**	**OR**	**95% CI**	**OR**	**95% CI**
**Immediate determinants**						
Women's low BMI	1.08***	1.05,1.12	1.04	0.96,1.13	1.34***	1.32,1.37
Maternal anemia	1.76***	1.72,1.81	--	--	--	--
Child diarrhea	1.13***	1.08,1.18	--	--	--	--
Child ARI	1.04	0.97,1.12	--	--	--	--
Women's weekly meat and fish consumption	0.83***	0.81,0.86	0.95	0.89,1.02	0.95***	0.93,0.97
Women's daily dark green vegetable consumption	0.99	0.96,1.02	0.98	0.92,1.05	0.99	0.97,1.00
**Nutrition and health interventions**						
At least 4 ANC visits	1.00	0.96,1.03	1.04	0.94,1.15	--	--
IFA consumption 100 tablets	0.97+	0.93,1.00	0.98	0.88,1.08	--	--
Deworming during pregnancy	0.98	0.94,1.02	0.89+	0.78,1.01	--	--
Weighed during pregnancy	0.89***	0.86,0.92	1.04	0.95,1.14	--	--
Pediatric full immunization	0.99	0.96,1.02	--	--	--	--
Pediatric vitamin A supplementation	0.98	0.95,1.01	--	--	--	--
Pediatric IFA	1.02	0.98,1.07	--	--	--	--
Pediatric deworming	0.95**	0.92,0.99	--	--	--	--
ICDS – food supplementation	0.94**	0.91,0.98	1.00	0.90,1.12	--	--
ICDS – health check-up	1.00	0.96,1.05	0.98	0.86,1.12	--	--
ICDS – health and nutrition education	1.02	0.98,1.07	1.18*	1.03,1.34	--	--
Using bednet	1.06***	1.04,1.09	1.06+	0.99,1.13	1.17***	1.15,1.20
**Underlying determinants**						
Number of children <5y	0.98***	0.97,0.98	1.15***	1.09,1.21	1.15***	1.13,1.16
Household SES index (0-10), score	0.84***	0.81,0.86	0.96***	0.95,0.98	0.98***	0.98,0.98
Improved sanitation facilities	0.93***	0.89,0.96	0.94	0.87,1.02	0.97**	0.95,0.99
Stool safe disposal	0.95**	0.92,0.98	0.85**	0.76,0.95	0.95*	0.92,0.99
Scheduled caste/tribe	1.06**	1.02,1.10	1.13**	1.04,1.22	1.08***	1.06,1.10
Hindu religion	1.03	0.97,1.11	1.02	0.88,1.18	1.04+	0.99,1.08
Muslim religion	1.11*	1.02,1.20	0.98	0.83,1.15	0.90***	0.86,0.95
Maternal schooling, y	0.97***	0.97,0.98	0.97***	0.97,0.98	0.99***	0.99,0.99
Married before 18 y	1.00	0.97,1.03	1.03	0.96,1.10	1.03***	1.01,1.05
Maternal age, y	0.99***	0.99,0.99	0.99**	0.98,1.00	1.00+	1.00,1.01

Other factors						
Male	1.00	0.98,1.03	--	--	--	--
Child aged 24-59 months	0.48***	0.46,0.49	--	--	--	--
Months of pregnancy	--	--	1.12***	1.10,1.14	--	--

BMI, Body mass index; ARI, Acute respiratory infection; ANC, antenatal care; IFA, iron and folic acid; ICDS, Integrated Child Development Services; SES, Social Economic Status.

P-values were obtained from multivariate linear regression models, adjusted for sampling weights:

***p<0.001, **p<0.01, *p<0.05, +p<0.10
